# Methodological pipeline for monitoring post-harvest quality of leafy vegetables

**DOI:** 10.1038/s41598-023-47873-4

**Published:** 2023-11-23

**Authors:** T. C. Tonto, S. Cimini, S. Grasso, A. Zompanti, M. Santonico, L. De Gara, V. Locato

**Affiliations:** 1 Department of Science and Technology for Sustainable Development and One Health, Unit of Food Science and Nutrition, Campus Bio-Medico University of Rome, Via Alvaro del Portillo 21, 00128 Rome, Italy; 2 Department of Science and Technology for Sustainable Development and One Health, Unit of Electronics for Sensor Systems, Campus Bio-Medico University of Rome, Via Alvaro del Portillo 21, 00128 Rome, Italy; 3 Department of Engineering, Unit of Electronics for Sensor Systems, Campus Bio-Medico University of Rome, via Alvaro del Portillo 21, 00128, Rome, Italy

**Keywords:** Plant sciences, Engineering

## Abstract

Plants are primary source of nutrients for humans. However, the nutritional value of vegetables tends to decrease once organ and tissue sinks are detached from the plant. Minimal processing of leafy vegetables involves cutting and washing before packaging and storage. These processing procedures result in stressful conditions and post-harvest disorders senescence-related can also occur. The aim of this work is to define a methodological pipeline to evaluate the “quality” changes of fresh cut leafy vegetables over their shelf-life. At this purpose, intra-species variability has been investigated considering two varieties of *Lactuca sativa* (var. *longifolia* and *capitata*), showing different susceptibility to browning. Since browning mainly depends on phenol oxidation, redox parameters as well as the activity of the enzymes involved in phenol biosynthesis and oxidation have been monitored over storage time. At the same time, the metabolic changes of the lettuce leaves have been estimated as response patterns to chemical sensors. The obtained sensor outputs were predictive of browning-related biological features in a cultivar-dependent manner. The integration of the results obtained by this multivariate methodological approach allowed the identification of the most appropriate quality markers in lettuce leaves from different varieties. This methodological pipeline is proposed for the identification and subsequent monitoring of post-harvest quality of leafy vegetables.

## Introduction

Nowadays, a diet rich in plant-based fresh foods is recommended to fit global nutritional claims, which points out the importance to consume low fat foods naturally rich in fibres, minerals and vitamins. In the actual social scenario, time to prepare food decreased and the intake of ready to eat foods based on minimal processed leafy vegetables increased the opportunity to reach the expected diet-dependent health benefits^1^. However, the shelf-life of fresh vegetables, already limited, can be even reduced after the washing, and cutting procedures required to produce ready to eat food. For this reason, packaging in modified atmosphere (MA) and controlled temperature are required to retard senescence and preserve the organoleptic and nutritional quality of fresh-cut vegetables over the estimated shelf-life. The assessment of ready to use food quality has been mainly determined relatively to microbial contaminations possibly occurring during producing chains^2^. In this context, electrochemical sensors have been employed to monitor water quality during washing steps and bacterial growth during storage^3^. However, depending on the genetic background, plant food products can also undergo post-harvest disorders, limiting shelf-life and/or consumers’ acceptability. In particular, browning disorder can affect leafy vegetables after manipulation especially at the wounding site. Browning mainly depends on the oxidation of plant phenols catalysed by the enzyme polyphenol oxidase (PPO) and their consequent polymerization in pigmented compounds. In intact tissues, phenols are localized in plant cell vacuole, whereas PPO is in the cytosol; after mechanical injuries, such as cutting procedures or wounding, the loss of the compartmentalization of cell components causes the interaction between PPO and phenols, which undergo oxidation^4,5^. Therefore, browning susceptibility could be directly related to phenol contents in plant tissues. Phenylalanine ammonia lyase (PAL) promotes the biosynthesis of phenolic compounds in response to different environmental stimuli by catalysing the conversion of phenylalanine in trans-cummaric acid^6^. In particular, PAL activity is induced by wounding^7^ and it has been demonstrated that treatments causing PAL inhibition reduced browning in fresh-cut vegetables^8,9^. Treatments of fresh-cut vegetables with reducing agents (such as ascorbic and citric acid) can also prevent browning occurrence, possibly protecting phenols from oxidation^10–12^. Moreover, the capacity of plant cells to defend themselves against oxidative events generally drops over storage time as consequence of senescence activation^13–15^. This might suggest that different genetic backgrounds can have a role in determining browning susceptibility based on endogenous antioxidant shield.

The aim of this study is to draw a methodological pipeline to identify quality indicators of minimal processed leafy vegetables that can be easily predicted by non-destructive technology. In this context, the multi-sensorial system could represent a real opportunity as a rapid tool for preliminary high-throughput screening thanks to its reduced time of analysis, low costs and the unnecessary employment of qualified personnel.

Since, quality indicators of fresh food can even be genotype-dependent, the analysis have been conducted on two different varieties of *Lactuca sativa*, a species sensitive to browning disorder: *Lactuca sativa* var. *capitata* (butterhead lettuce) and *Lactuca sativa* var. *longifolia* (romaine lettuce). Indeed, investigating intra-species variability for a phenotype parameter, allows to restrict genetic background inference, as well as it gives the opportunity to finely test the methodological approach. Over shelf-life time, browning index, as well as photosynthetic pigment levels, have been estimated in the selected lettuce varieties. At the same time, the trend of redox related features, such as antioxidant capacity, total phenol content, PPO and PAL activities have been also determined. Electrochemical-based sensors, previously tested to monitor ready to use food quality in terms of microbiological safety^3^ associated to a gas sensor array have been employed to monitor changes occurring in lettuce varieties during shelf-life period. The innovative analytical approach based on the combined analysis of solutes and volatile molecules released from leafy vegetables during shelf-life allowed to monitor more accurately the multiplicity of chemical and biochemical transformations undergone by the food during its physiological spoilage. Therefore, the pattern of the responses obtained by sensor analysis have been correlated with quality indicators emerging from the performed chemical analysis. Finally, the correlation between different browning phenotypes and redox markers, as quality indicators, and sensor response has been validated.

## Materials and methods

### Plant material

Commercially ready to eat leafy vegetables from two varieties of *Lactuca sativa* (var. *longifolia* and *capitata*, named romaine lettuce and butterhead lettuce, respectively), were obtained from local market and stored at 4 °C. Batches of ready to eat products with a 5-day shelf-life were selected and purchased, considering the expiration day as day 5 of storage. For each sample, two packages from the same batch were analyzed: one package was opened on day 1, acclimated under air conditions for 4 h before analysis, and stored at 4 °C until the expiration day, while the other package was opened on the expiration day after storage at 4 °C. The analyses were conducted daily from day 1 (the day the package was opened) until day 5 (the expiration day) for the opened package (air condition) and only on day 5 for the closed package stored at 4 °C until being used (MA condition). MA composition for lettuce is: 85–90% N_2_; 5–10% CO_2_; 1–3% O_2_ according to recommendation^16^.

### Dry weight determination

Every day, 1 g of sample was weighed for 5 biological replicates from the same bag, and its weight was measured daily during storage in a 60 °C oven until it did not change. The dried weight was calculated as a percentage of the initial fresh weight.

### Photosynthetic efficiency determination

The photochemical efficiency of PSII (PhiPSII) was determined by the LI-600 Porometer/Fluorometer^17^. The photosynthesis efficiency of lettuce leaves was measured every day of shelf-life period (from day 1 to day 5) under air conservation and only on day 5 after MA storage.

### Leaf imaging processing and browning index calculation

A color scale in RGB (Red, Green, Blue) was obtained through acquisitions using a chromameter, using the set-up previously described^18^. The RGB scale was converted in CIE L*A*B* value by a Software Photopea. The L* parameter represented the brightness or grayscale of the color with a range between 0 (black) and 100 (white). The a* parameter represented the red-green axis of the color with a range of negative and positive values, where a negative value indicates a green hue and a positive value indicates a red hue. The b* parameter represented the yellow-blue axis of the color. It also had a range of negative and positive values, where a negative value indicates a blue hue and a positive value indicated a yellow hue. With these parameters, three pieces of information were obtained for the browning index^19^, the hue angle (position of the color within a color wheel)^20^, the Whiteness index (WI) and the total color different (ΔE)^21^.

### Determination of pigment levels

0.2 g of leaf was weighed and pulverized using liquid nitrogen, and 80% acetone was added in a 1:3 weight to volume (w:v) ratio. The mixture was homogenized using a pestle and then centrifuged under relative centrifuge force (rcf) of 14,000×*g* for 10 min at 4 °C. The supernatant containing the liposoluble pigments was recovered. The absorbance was measured by spectrophotometer UV-1800 Shimidazu, at the peak wavelengths: 663 (chlorophyll a; Ca), 646 (chlorophyll b; Cb), 470 (carotenoids; Cx-c) nm. The results were expressed as mg kg^−1^ of lettuce fresh weight^22^.

$${\text{Ca }} = { 12}.{25 } \times {\text{ ABS}}_{{{663}}} {-}{ 2}.{55 } \times {\text{ ABS}}_{{{648}}} ,$$

$${\text{Cb }} = { 2}0.{31 } \times {\text{ ABS}}_{{{648}}} {-}{ 4}.{91 } \times {\text{ ABS}}_{{{663}}} ,$$

$${\text{Cx-c }} = \, \left( {{1}000 \, \times {\text{ ABS47}}0 \, - { 1}.{82 } \times \, \left[ {{\text{Ca}}} \right]} \right) \, - { 85}.0{2 } \times \, \left[ {{\text{Cb}}} \right])/{198}.$$

### Antioxidant capacity assays

For all antioxidant assays, the same sample preparation was used: 0.3 g of fresh leaf tissue was powdered in liquid nitrogen and homogenized in 100% EtOH with a w:v ratio of 1:6. The mixture was then centrifuged at 14,000×*g* (rcf) for 10 min at 4 °C, and the resulting supernatant was collected. For the experiments, three biological replicas were obtained, and for each biological replica, there were made three technical replicas. All the results have been normalized for kg of lettuce fresh weight.

DPPH (1,1-Diphenyl-2-Picrylhydrazyl) radical scavenging assay was analyzed as previously reported^23^ with some modifications. 20 μL of the supernatant was incubated with 180 μL of a 0.1 mM DPPH solution for 20 min in the dark. Two absorbance readings were taken: one at time zero and another after 20 min of incubation using a microplate reader (TECAN Infinite M200PRO) at 518 nm. Trolox (6-hydroxy-2,5,7,8-tetramethylchroman-2-carboxylic acid) was used as a standard for calibration curve construction in the range of 20–200 mg L^−1^, and the results were expressed as mmol Trolox equivalent kg^−1^.

FRAP (ferric-reducing antioxidant power) assay was based upon the methodology previously described^24^. Briefly, 10 μL of the supernatant was incubated with 190 μL of the FRAP reagent consisting of ACETATE BUFFER/TPTZ/Fe (in a ratio of 10:1:1). The absorbance values were read using a microplate reader (TECAN Infinite M200PRO) at 593 nm after 6 min of incubation. Trolox (6-hydroxy-2,5,7,8-tetramethylchroman-2-carboxylic acid) was used as a standard for calibration curve construction in the range of 20 mg L^−1^, and the results were expressed as mmol Trolox equivalent kg^−1^.

TEAC (Trolox equivalent antioxidant capacity) assay was evaluated as previously described^25^. Briefly, 20 μL of the sample were incubated with 180 μL of ABTS^⋅+^ solution (7 mM ABTS and 2.5 mM potassium persulfate) for 20 min in the dark. At time zero and after 20 min of incubation, the absorbance readings were taken using a microplate reader (TECAN Infinite M200PRO) at 734 nm. Trolox (6-hydroxy-2,5,7,8-tetramethylchroman-2-carboxylic acid) was used as a standard (20–100 µM) for calibration curve construction, and the results were expressed as mmol Trolox equivalent kg^-1^.

### Total phenolic content determination

Total phenolic content (TPC) was evaluated using the Folin–Ciocalteau method^26^. 20 μL of the supernatant was suspended in 1580 μL of 50% EtOH. Then, 100 μL of Folin reagent was added to both the samples and standards, and they were incubated in the dark for 8 min. After the incubation, 300 μL of Na_2_CO_3_ (at a concentration of 0.2 kg L^−1^) was added, and a second incubation of 2 h was performed. At the end of the incubation time, the samples were centrifuged at room temperature for 5 min. Finally, the absorbance was measured using a microplate reader (TECAN Infinite M200PRO) at a wavelength of 765 nm. Concentration of total phenolic compounds were expressed in g of gallic acid equivalents per kg^−1^ of lettuce fresh weight.

### Determination of enzyme activity

The spectrophotometric assays for determining enzymatic activities were performed by using the spectrophotometer UV-1800 Shimidazu. Enzyme activity has been normalized per kg protein. Protein content was measured using the Biorad^®^ Protein Reagent with bovine serum albumin (BSA) as a standard. Three tubes were prepared for each sample and the following reagents were added: 790 µL of deionized water, 10 µL of sample and 200 µL of Biorad^®^Protein Assay Dye Reagent Concentrate. The tubes, containing the reaction mixture, were vortexed and incubated at room temperature for 15 min to allow the dye to bind proteins. The spectrophotometer reading was recorded at maximum wavelength absorption of 595 nm^27^.

### PAL activity

The activity of Phenylalanine Ammonia-Lyase was measured as previously described^28^. Samples (2 g) were homogenized at 4 °C with 16 mL of 50 mM borate buffer (pH 8.5) containing 5 mM 2-mercaptoethanol and 0.2 mM of polyvinylpyrrolidone. The homogenate was filtrated through 4 layers of Miracloth and centrifuged under rcf of 20,000×*g* at 4 °C for 20 min. After the addition of 550 µL of 50 mM l-phenylalanine to the supernatant, the obtained mixture was incubated at 40 °C for 1 h. The absorbance was measured at 290 nm before and after incubation. Results were expressed as mol cinammic acid h^−1^ kg^−1^ protein.

### PPO activity

The activity of Polyphenol Oxidase has been measured as previously described^28^ with some modifications. Samples (4 g) were homogenized with 12 mL 50 Mm Na-phosphate buffer (pH 6.5). The homogenate was filtered and centrifuged under rcf of 20000xg for 20 min. A volume of extract of 0.75 mL was incubated with 0.3 mL of 0.1 Mm caffeic acid ethanolic solution and the absorbance was recorded at 480 nm in kinetic configuration for 5 min. Results were expressed as mol oxidized phenols s^−1^ kg^−1^ protein.

### Liquid sensor platform

To assess electrochemical analysis of ready-to-eat salad, a liquid analysis sensor, the BIONOTE for Liquids (BIONOTE-L), was used^3^. Its working principle is based on the cyclic voltammetry method, which has already been used for the analysis of various food matrices^29,30^. In particular, the device consists of two parts: an electronic interface, which provides an input signal (a triangular voltage waveform from + 1 V to − 1 V) and registers data at the output, and a screen-printed electrode probe (SPE; DRP-250BT, Metrohm; Herisau; Switzerland) as a disposable sensor terminal. The applied input voltage induces redox reactions of the addressable molecules when the electrode is placed in contact with the solution. Consequently, the electrons involved in these reactions generate an output current, which is detected. The frequency of the input signal was 10 mHz while the output was acquired at 40 ms intervals. As a result, the multi-dimensional data output was analysed using a fingerprinting approach. Samples were analysed according to the following experimental setup. Briefly, 2 g of ready-to-eat lettuce were poured into a glass beaker containing 20 mL of distilled water and were let to incubate at 20–22 °C (room temperature; RT) for 30 min, while gently stirring every 30 s. Finally, a volume of 3 mL was withdrawn from the soaking solution, and it was analysed by the sensor.

### Gas sensor platform

The used gas sensor platform is a custom-made device that exploits an array of commercial MOX sensors from Figaro (Figaro Engineering Inc., Japan). Specifically, the used sensors are the following: TGS2602, TGS2610, TGS2600, TGS2620. The MOX sensors are biased using the circuit layout suggested by the datasheets: the implemented electronic interface is a voltage divider. The internal heater of each sensor is powered as well. The output signal produced by each voltage divider is acquired by a 12-bit ADC with a resolution equal to 1.22 mV. Thus, the platform produces a data array as output, equal to the output voltages of the sensors, with a sampling frequency of 1 Hz. The gas sensor platform was connected to a sealable measuring chamber and the head space was collected through a controlled closed pneumatic circuit. Samples were analysed according to the following experimental setup: 2 g of ready-to-eat lettuce were placed into the measuring chamber before it was sealed, then, samples were let to incubate at RT for 20 min in order the head space to be formed; finally, the head space was analysed by the gas sensor platform for 180 s and the difference between the starting and final voltage output was calculated. After each measure, the gas sensor platform was let to clean for 720 s using room air.

### Statistical analysis

All statistical analyses were performed with GraphPad Prism 8 software (GraphPad Software, San Diego, CA, USA). One-way ANOVA followed by Tukey test correction was performed. p < 0.05 were set as the significance cut-off. All values were presented as means ± SE. To get a simplified representation of the sensors’ multidimensional data set, multivariate data analysis techniques were applied. Principal Component Analysis (PCA) and Partial Least Square (PLS), were performed using PLS-Toolbox (Eigenvector Research Inc., Manson, WA, USA) in the Matlab Environment (The MathWorks, Natick, MA, USA).

## Result and discussion

### Image processing for phenotyping butterhead lettuce and romaine lettuce under air and MA storage

*Lactuca sativa* is a plant species which generally undergoes browning during fresh cut leaves’ storage period. However, intra-species variability occurs allowing the identification of cultivars with contrasting susceptibility to browning.

At this purpose, two different cultivars of *Lactuca sativa* (var. *capitata* and *longifolia*), named butterhead lettuce and romaine lettuce, were selected given their significant difference in quality deterioration during storage under low temperature throughout a storage period of 5 days (approximately 10 days after cutting). The overall butterhead lettuce and romaine lettuce quality was monitored in terms of enzymatic browning and leaf senescence during storage under air and under modified atmosphere (MA), which composition is reported in “Materials and methods” section.

During the considered storage period, no alterations of general parameters related to leaf quality were observed. In particular, dry weight (and complementarily water content) of butterhead and romaine lettuce leaves were similar at the starting point of analysis (6.3 ± 1.2% and 6.2 ± 1.1% at day 1, respectively) and they did not change over storage period in both cultivars (5.6 ± 1.3% and 4.8 ± 1.1% at day 5, respectively). No variation of parameters related to leaf physiology such as photochemical efficiency of photosystem II (PhiPSII) was also observed under air storage; whereas a decrease of PhiPSII was registered on day 5 for leaves stored under MA (5* samples) compared to samples stored under air from day 1 to day 5 (Fig. S1). MA is mainly characterized by reduced oxygen level with the aim to restrain catabolic events as well as eventual microbial growth. It has been reported that low oxygen can inhibit photosynthesis^31^. To explain this physiological effect due to low oxygen exposure, different explanations have been proposed, such as starch biosynthesis or sugar allocation inhibition^32^. However, in detached leaves low oxygen dependent photosynthesis inhibition could be mainly due to a reduction of respiratory losses from leaves with consequent drop in the utilisation of photo-assimilates, whose accumulation can act as negative feedback on photosynthesis^33,34^.

To quantify leaf quality deterioration and browning, three colouring parameters, L*, a* and b* values, were monitored in butterhead lettuce and romaine lettuce leaves (Table S1).

a* value is a colour parameter related to browning and to the breakdown of chlorophylls^12,35^. The increase of this parameter indicates a shift from greenness to redness. In butterhead lettuce, a* values started to increase from day 4 of storage and increased up to 40% on day 5 of storage, while the observed increase in the MA samples accounted for 30% (Table S1). Whereas in romaine lettuce, only a 13% increase was observed on day 4 of storage and 28% on day 5 of storage in air. At the end of storage under MA the lowest a* value was obtained for romaine lettuce leaf (7%) (Table S1). Despite a similar genetic background, the higher increase of a* value observed in butterhead lettuce compared to romaine lettuce underlies the highest sensitivity of this cultivar to browning upon storage.

L* value, a parameter related to sample lightness and chlorosis^12,36^ decreased with time both in butterhead lettuce and romaine lettuce during storage. However, this decrease was significantly lower (p < 0.05) in romaine lettuce (5% decrease) than in butterhead lettuce (11% decrease) on day 5 of storage (Table S1). Whiteness has been widely used by researchers as an indicator of vegetable deterioration and is strictly related to L* values^12,37^ Coherently to L* values, the whiteness decreased of 18% during cold storage in butterhead lettuce and by 5% in romaine lettuce.

**Figure 1:**
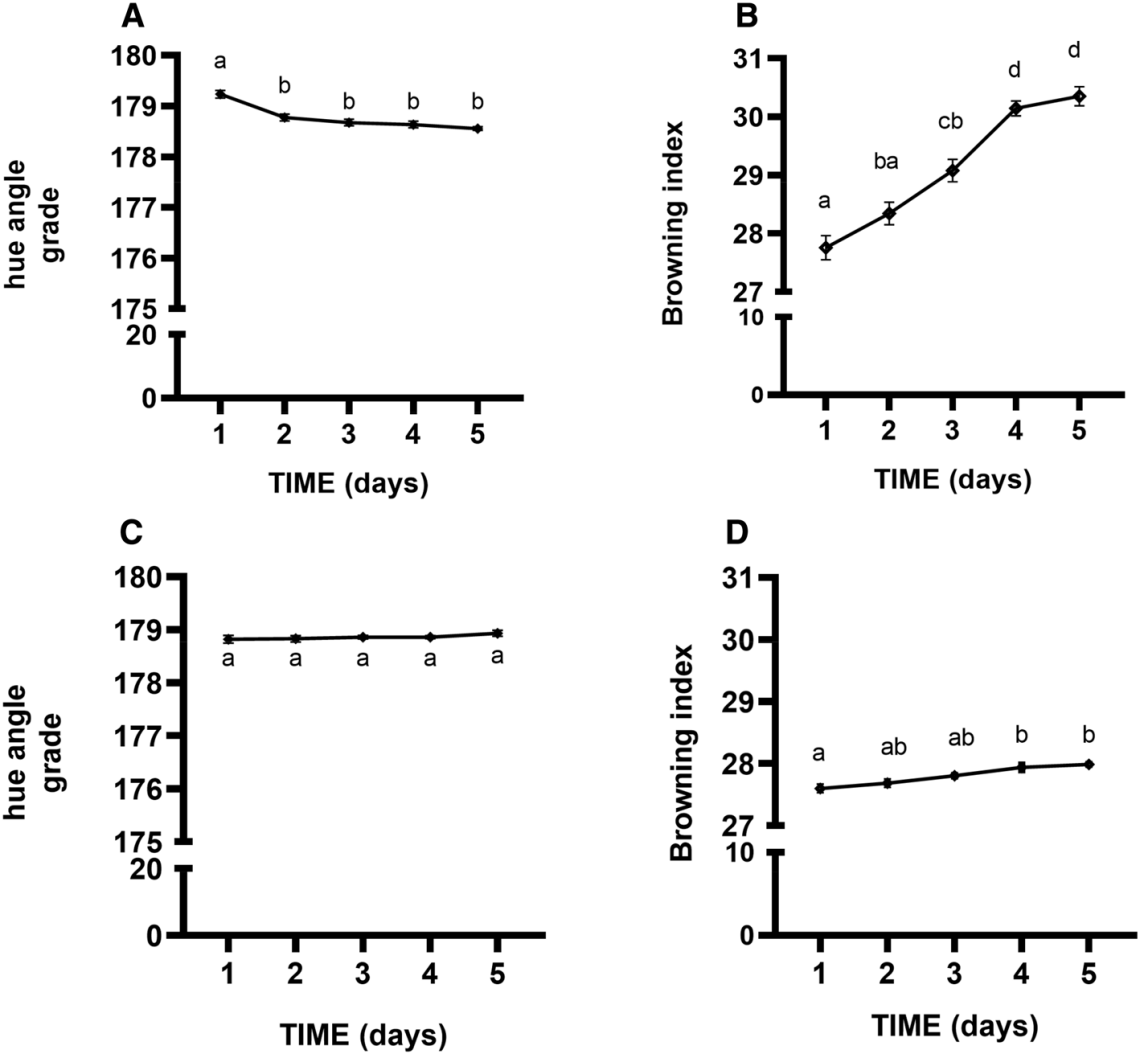
Hue angle grade and browning index of butterhead lettuce (**A,B**) and romaine lettuce (**C,D**) during storage period under air conditions. Values are means ± SE of at least three biological replicates, each one with three technical replicates. Different letters indicate a statistical difference (p < 0.05) based on one-way ANOVA followed by Tukey test correction.

This is reflected in a significantly lower hue angle in butterhead lettuce (p < 0.05) thus confirming the high capacity of romaine lettuce to maintain higher overall leaf quality (Fig. 1A–C). The browning index increased over time only in butterhead lettuce stored under air conditions (Fig. 1B–D). In the MA storing conditions the browning index was estimated 30.5% for butterhead lettuce and 27.5% for romaine lettuce. Total colour difference (ΔE), which is a combination of parameters L*, a* and b* values, is a colorimetric parameter extensively used to characterize the variation in colours depending on storage conditions. Here, ΔE values were calculated as colour variation during sample conservation. The ΔE value tended to increase during storage. The increasing ΔE value was lower in romaine lettuce stored in air than in butterhead lettuce, 5.57 and 14.07 respectively, showing that romaine lettuce is more efficient in maintaining fresh colour after cutting.

### Pigment content of butterhead lettuce and romaine lettuce under air and MA storage

**Figure 2:**
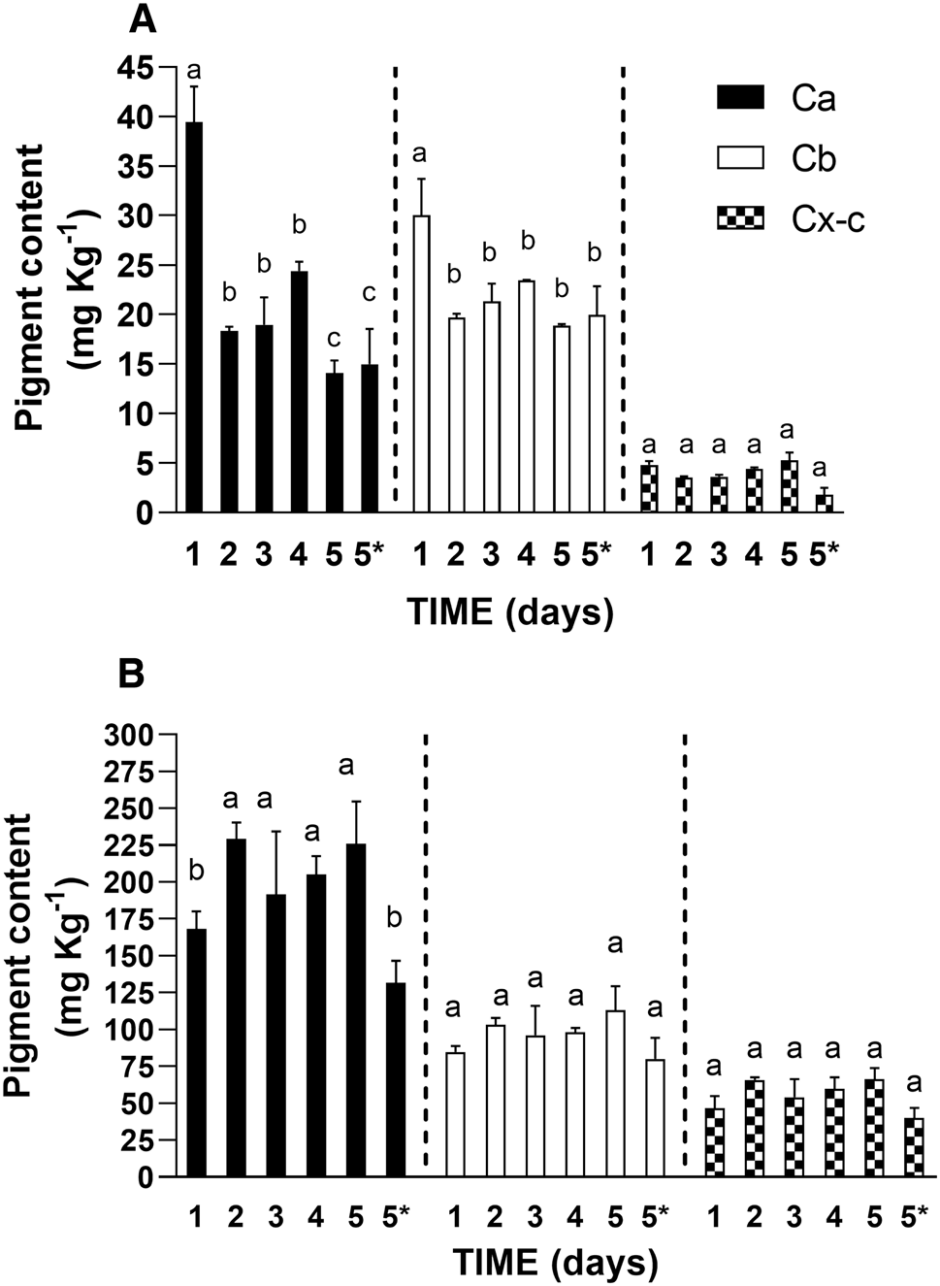
Photosynthetic pigments of butterhead lettuce (**A**) and romaine lettuce (**B**) during storage period under air and MA. Values are means ± SE of at least three biological replicates, each one with three technical replicates. Different letters indicate a statistical difference (p < 0.05) based on one-way ANOVA followed by Tukey test correction. 5* indicates the data referred to the samples collected at day 5 of storage under MA condition. *Ca* chlorophyll a, *Cb* chlorophyll b, *Cx-c* carotenoids.

Detached leaves obviously undergo nutrient deficiency, which is related to leaf senescence activation^36^. In this context, chlorophyll breakdown generally occurs^37,38^. However, image processing clearly showed that butterhead lettuce is more susceptible to de-greening than romaine lettuce over storage conditions. It is known that during food processing and storage, almost all green vegetables lead to chlorophylls degradations and that the extent of this degradation depends on the considered plant species and varieties^39^. In order to evaluate the contribution of chlorophyll breakdown to browning, the level of photosynthetic pigments was evaluated. As shown in Fig. 2A, a significant decrease (p < 0.05) in chlorophyll contents during storage was observed in butterhead lettuce. Indeed, in butterhead lettuce, a strong reduction in chlorophyll a and chlorophyll b contents were observed starting from day 2 to the endpoint of the storage time. This reduction was not observed in romaine lettuce (Fig. 2B) in which pigment levels did not change till the end of the storage under both air and MA conditions. The variation of the content of these pigments was reflected in a chlorophyll a/b ratio significantly lower in butterhead lettuce than in romaine lettuce at the endpoint of the storage time, with values of 0.74 and 3.01 in butterhead lettuce and in romaine lettuce, respectively (p < 0.05). No significant differences were observed among butterhead lettuce samples stored under air and MA conditions (Fig. 2).

### Antioxidant capacity of butterhead lettuce and romaine lettuce under air and MA storage

**Figure 3:**
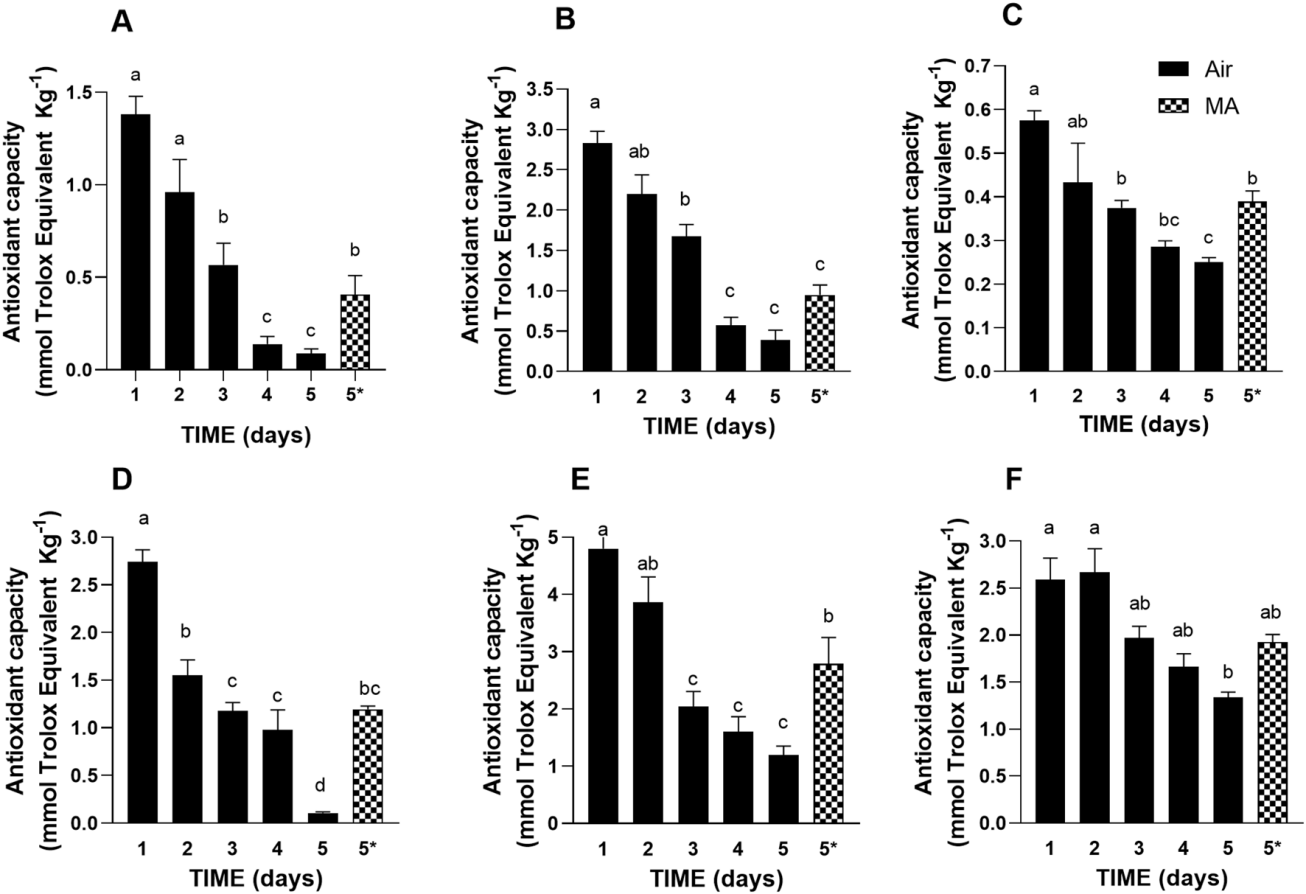
Antioxidant capacity of butterhead lettuce (**A–C**) and romaine lettuce (**D–F**) over storage period under air and MA. (**A**) DPPH scavenging capacity of butterhead lettuce leaves; (**B**) FRAP scavenging capacity of butterhead lettuce leaves; (**C**) TEAC scavenging capacity of butterhead lettuce leaves; (**D**) DPPH scavenging capacity of romaine lettuce leaves; (**E**) FRAP scavenging capacity of romaine lettuce leaves; (**F**) TEAC scavenging capacity of romaine lettuce leaves. Values are means ± SE of at least three biological replicates, each one with three technical replicates. Different letters indicate a statistical difference (p < 0.05) based on one-way ANOVA followed by Tukey test correction. 5* indicates the data referred to the samples collected at day 5 of storage under MA condition.

Since oxidative events are mainly involved in senescence and wounding^40,41^, promoting browning, the cell capacity to defend its components from oxidation was evaluated in the two cultivars. At this purpose, in this study we monitored the antioxidant power by using FRAP, TEAC and DPPH assays. The simultaneous use of different antioxidant capacity assays is a good experimental practice since each method can show different sensitivity toward different antioxidants^42,43^. This depends on the fact that each antioxidant molecule consists in a redox couple (reduced form/oxidized form) with a redox potential describing the molecular tendency to donate electron(s)^44^. This attitude is also strongly influenced by the acceptor’s redox potential and, therefore, by the chemical nature of the radical used in the assay^45,46^. The romaine lettuce antioxidant capacity is significantly higher than that of butterhead lettuce when measured by all antioxidant assays (p < 0.05; Fig. 3). During sample storage in air, the antioxidant capacity reached lower levels at the end of shelf-life compared to the starting point both in romaine lettuce and in butterhead lettuce (Fig. 3). The antioxidant capacity measured by the three abovementioned assays showed the same trend of decrease that is characterized by an antioxidant capacity that is already significantly reduced at day 3 of cold storage in air (p < 0.05). The sample storage in MA reduced this decrease in antioxidant capacity, which therefore remained higher at the endpoint of conservation time in both butterhead lettuce and romaine lettuce (Fig. 3). Since all antioxidant capacity assays are based on the capacity of antioxidants to reduce a radical, these methods are able to measure only the active (reduced) form(s) of antioxidants and not the already oxidized form(s). Under low oxygen environment, a reduction in oxidative events could be expected. However, photosynthesis was still active in the leaves stored under MA (Fig. S1), and oxygen produced by leaves can partly reduce the “protective effect” of MA, coherently with a moderate reduction of antioxidant capacity of the MA samples compared to the stronger one observed for air stored samples over the same monitored storage period (Fig. 3).

### Phenol metabolism of butterhead lettuce and romaine lettuce under air and MA storage

**Figure 4:**
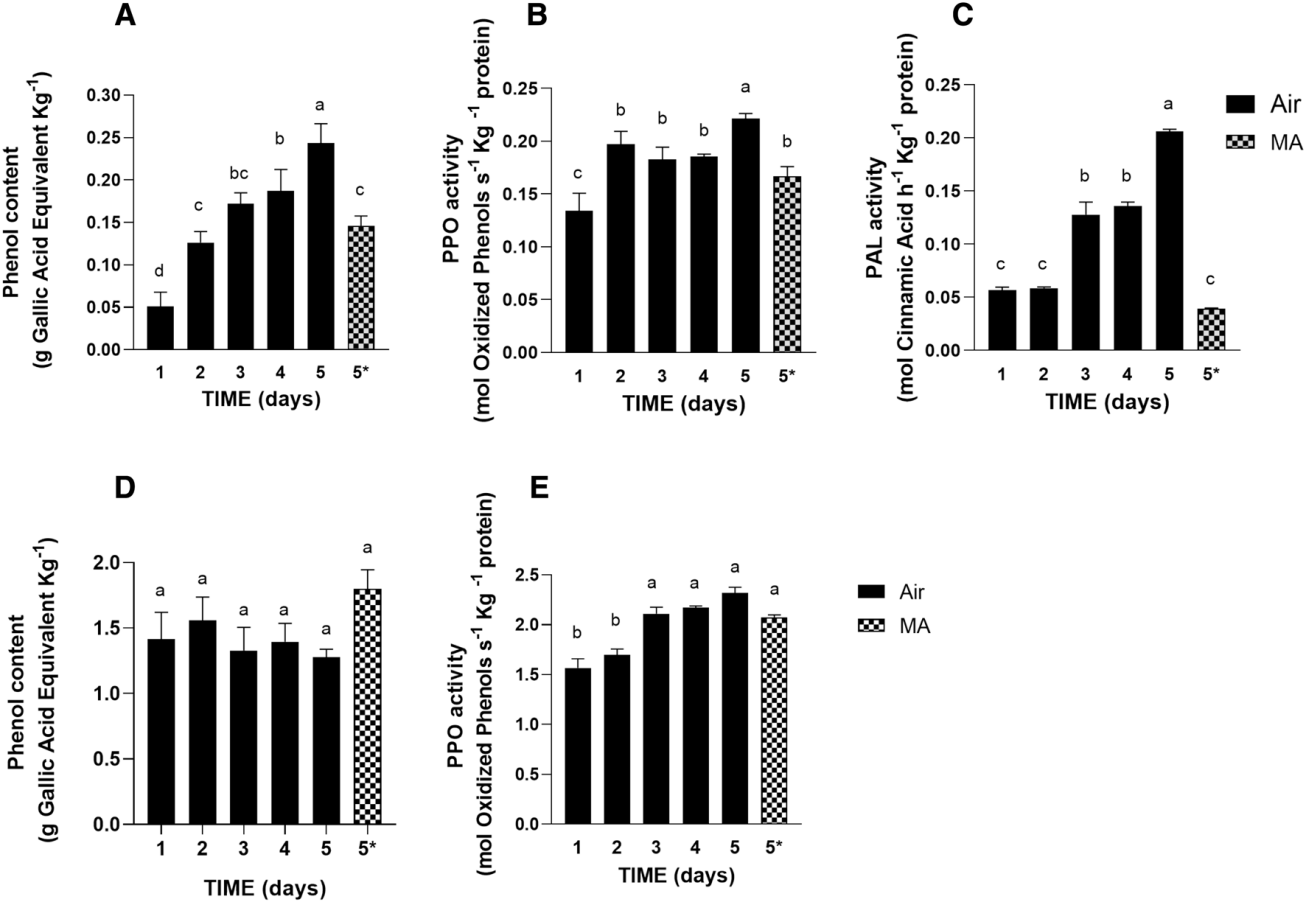
Phenol metabolism of butterhead lettuce and romaine lettuce under air and MA storage conditions. Total Phenol Content (TPC) in butterhead lettuce (**A**) and in romaine lettuce (**D**). Polyphenol oxidase (PPO) activity in butterhead lettuce (**B**) and in romaine lettuce (**E**). Phenylalanine ammonia lyase (PAL) activity in butterhead lettuce (**C**). Values are means ± SE of at least three biological replicates, each one with three technical replicates. Different letters indicate a statistical difference (p < 0.05) based on one-way ANOVA followed by Tukey test correction. 5* indicates the data referred to the samples collected at day 5 of storage under MA condition.

It has been reported that the main antioxidant compounds present in lettuce leaves are phenols^47^. Therefore, it has been expected that the measured antioxidant capacity could mainly depend on the level of these compounds. Consistently, leaves from romaine lettuce, characterized by higher total antioxidant capacity compared to butterhead lettuce ones (Fig. 3), also showed constitutive higher levels of total phenolic content (TPC) than butterhead lettuce (Fig. 4A–D).

TPC was influenced in butterhead lettuce during storage in air while no statistically significant differences have been monitored in romaine lettuce. In particular, under air conditions a 150% increase of TPC was registered in butterhead lettuce leaves starting from day 2 of storage (Fig. 4A). This increase is reduced by MA storage. Whereas, in romaine lettuce, the TCP was not affected during the storage period and not depended on whether stored in air or in MA (Fig. 4D).

Since browning mainly depends on phenol oxidation extent, the activity of polyphenol oxidase (PPO) was monitored in all described experimental conditions. At the starting of the storage period, PPO activity is about 10 times higher in romaine lettuce than in butterhead lettuce (Fig. 4B–E). Moreover, in butterhead lettuce, the activity of this enzyme increased starting from day 2 of storage until the end of the storage period in air conditions. After 5 days under MA conditions (sample 5*) the induction of PPO activity was slightly reduced compared to the samples collected at day 5 after air storage (Fig. 4B). In romaine lettuce, PPO activity showed a similar response to air storage although with a different timing. Indeed, PPO activity started to increase from day 3 of conservation and then remained constant throughout the storage time (Fig. 4E). Therefore, the observed different regulation of PPO activity in romaine lettuce and butterhead lettuce mirrored these cultivars’ contrasting susceptibility to browning (Fig. 1).

The trend of the activity of the first phenol biosynthetic enzyme, phenyl-alanine ammonia lyase (PAL), was also followed until the endpoint of the storage period. In butterhead lettuce PAL activity significantly increased from day 3 after air storage and continues to increase until the end of the storage (p < 0.05; Fig. 4C). Interestingly, the induction of PAL activity was repressed by the storage in MA, consistently with the TPC increase reduction observed under this storage condition compared to air storage situation (Fig. 4A). In romaine lettuce, undetectable levels of PAL activity were observed from the beginning until the end of the storage in air as in MA. As previously reported, the different regulation of PAL activity also correlated with a different sensitivity to stress of the analyzed varieties, since PAL is generally induced by wounding and mechanical injuries^7^. Despite the different regulation of phenol biosynthesis observed in both cultivars, the antioxidant capacity dropped in both cultivars over storage period (Fig. 3), suggesting that the new produced phenols were promptly oxidized in butterhead lettuce and consequently not more reactive as radical scavengers.

In contrast with previous reports^5,6^, the results obtained in this study showed that browning susceptibility was not obviously related to phenol content and/or their oxidation in leafy vegetables, since romaine lettuce showing lower tendency to browning than butterhead lettuce (Fig. 1B–D), also presented higher TPC and PPO activity (Fig. 4).

### Sensor analysis of butterhead lettuce and romaine lettuce under air and MA storage

**Figure 5:**
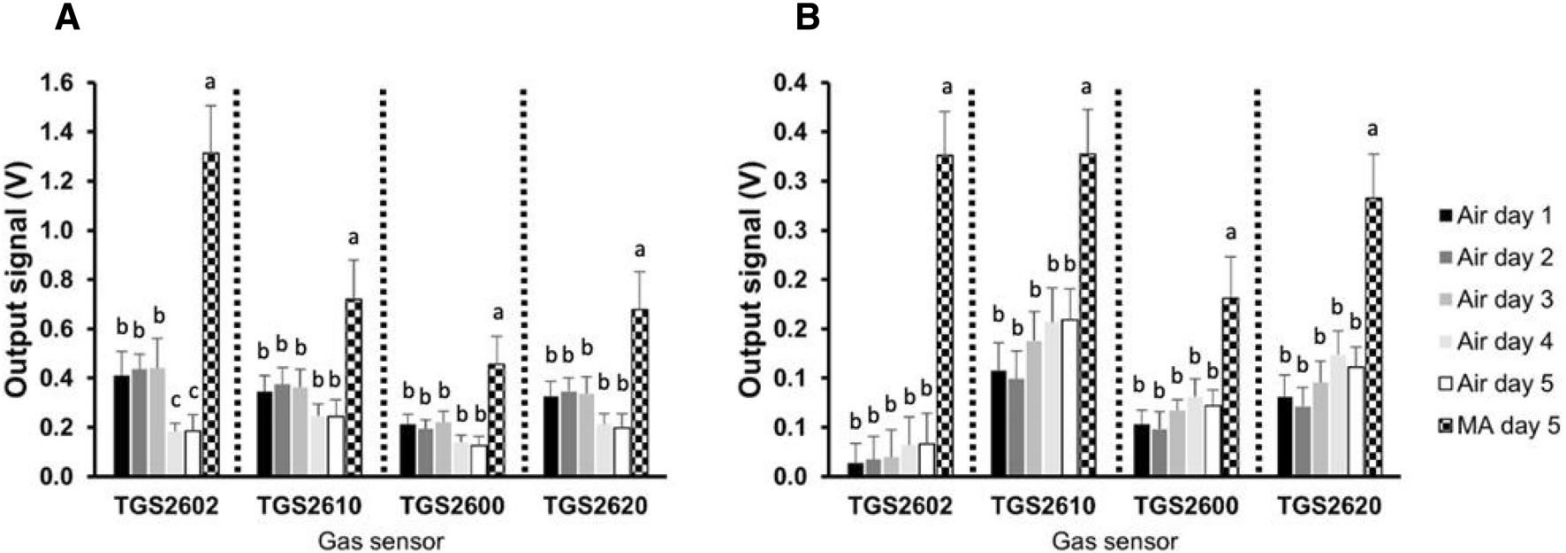
Gas sensor array analysis of butterhead lettuce (**A**) and romaine lettuce (**B**) headspace during storage period under air and MA conditions. Values are means ± SE of at least three biological replicates, each one with three technical replicates. Different letters indicate a statistical difference (p < 0.05) based on one-way ANOVA followed by Tukey test correction.

In this study, a not destructive method has been employed to evaluate quality changes identified by metabolic features related to post-harvest disorders, such as browning, in minimal processed leafy vegetables under the described experimental conditions. In particular, the head space of butterhead lettuce and romaine lettuce leaves have been monitored by a gas sensor array consisting of 4 sensors (as described in “Materials and methods” section) over storage period. The gas sensor array platform features low power consumption and long operating life. Despite these sensors are commonly used in the detection of gaseous air contaminants for indoor air quality monitoring and for biomedical applications^48^, they have never been used for food quality assessment. Therefore, their high sensitivity to hydrogen, ethanol, methane, iso-butane, propane and other odorous gases such as ammonia and hydrogen sulphide makes them proper candidate for food odours fingerprinting. The gas sensor array’s obtained fingerprints showed slight differences for the two analysed cultivars. In particular, in butterhead lettuce only the output signal obtained from TGS2602 gas sensor array significantly decreased at day 4 of shelf-life (p < 0.05); whereas in romaine lettuce any change in the intensity of the signals were recorded. Under MA conditions, the output values were always higher than those registered under air conditions (Fig. 5). Since MA samples have been immediately analyzed after package opening, it can be supposed that for these samples, the sensors underwent to up-stimulation since the analysed headspaces were enriched of the gas accumulated under MA storage conditions or released by oxidative metabolism re-activation^49,50^.

In the same experimental conditions, the electrochemical fingerprints of butterhead and romaine lettuce have also been obtained by the liquid sensor as described in material and methods section. The BIONOTE-L liquid sensor has already been used in some studies to assess water quality and food safety^3,51^ and also for biomedical applications^51,52^. This novel system has three peculiar features in comparison to the standard approach: it uses bare screen-printed electrodes, the signal is highly stable and low-noise due to the electronic interface, and finally, it allows to modulate the frequency and amplitude of the input signal.

**Figure 6:**
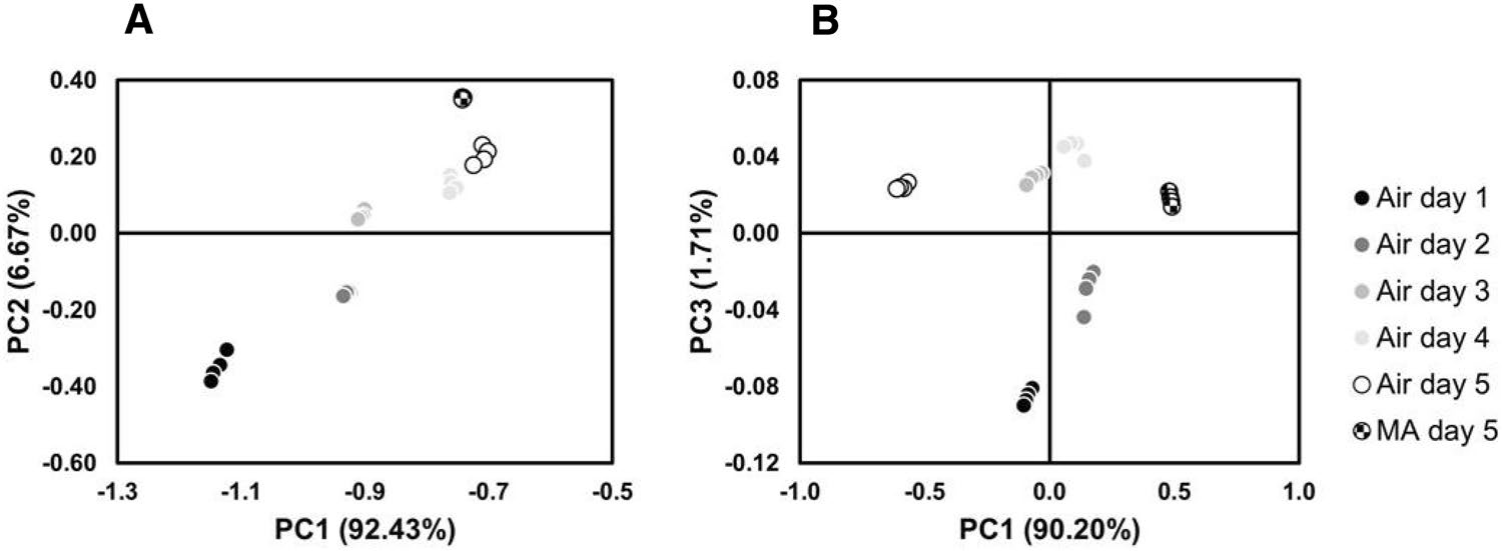
Scores plot of a Principal Component Analysis (PCA) showing the distribution of butterhead lettuce (**A**) and romaine lettuce (**B**) over storage period along the main principal components (PC1, PC2 and PC3).

The obtained multi-dimensional data outputs of liquid sensor were analysed by Principal Component Analysis. The scores plot of the first two Principal Components (PCs) in Fig. 5A, accounting for about 99% of the explained variance, highlighted the formation of 6 distinct clusters along the PC1 for butterhead lettuce. Each cluster included the outputs derived from the samples collected at a certain experimental time point. Therefore, by electrochemical analysis butterhead lettuce samples collected at different time points can be clearly discriminated during shelf-life period. It is also notably that the outputs registered at day 5 of shelf-life from the samples stored under MA were discriminated even if their cluster was closed to that one representing the samples collected at the same time under air conditions (Fig. 6A). On the other hand, the scores plot of the two PCs in Fig. 6B, accounting for about 92% of the explained variance, allowed to clearly distinguish the 2 clusters of AM and air samples at day 5 of shelf-life along the PC1 for romaine lettuce; whereas the 4 clusters relative to the romaine lettuce samples collected from the day 1 to day 4 of shelf-life are mainly discriminated along PC3 (Fig. 6B).

**Figure 7:**
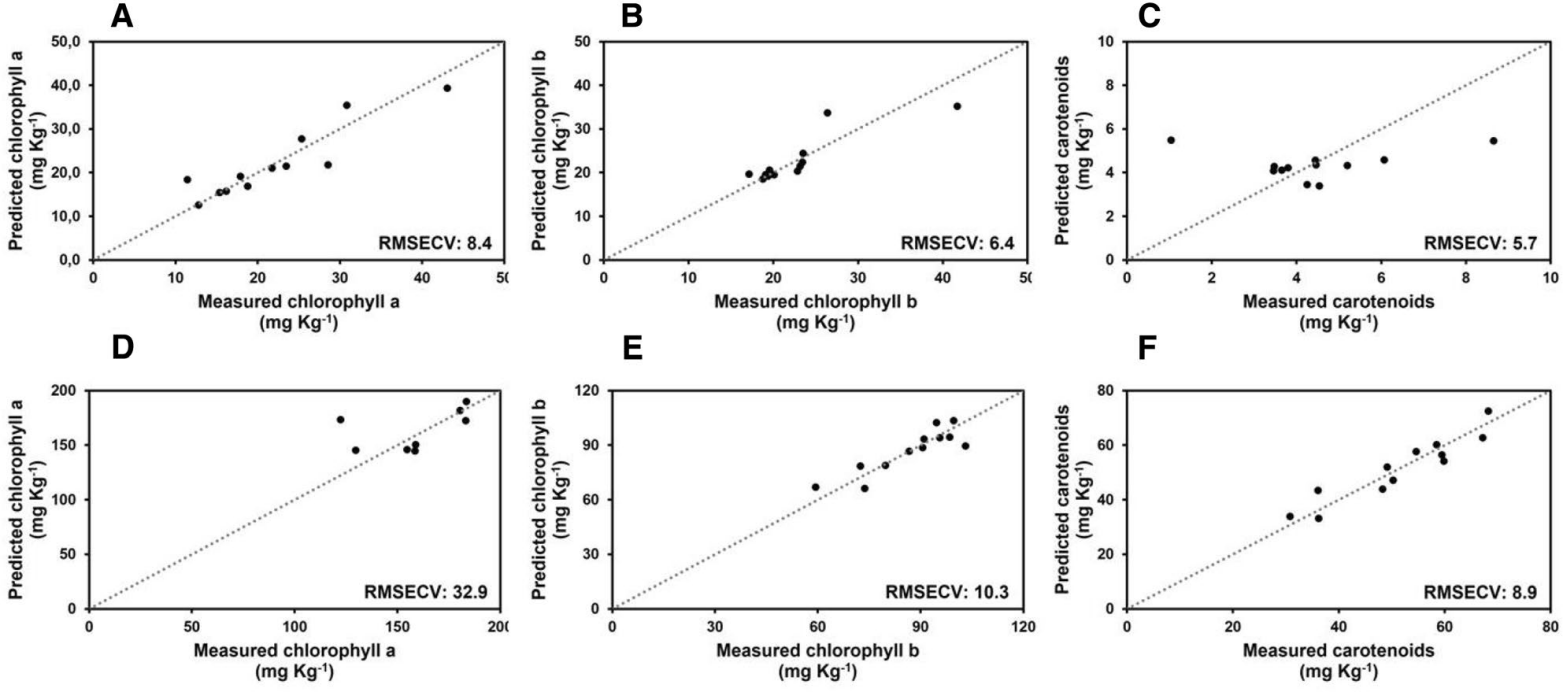
Calculated partial last square model for the prediction of photosynthetic pigments on butterhead lettuce (**A–C**) and romaine lettuce (**D–F**). RMSECV associated with the models are reported.

**Figure 8:**
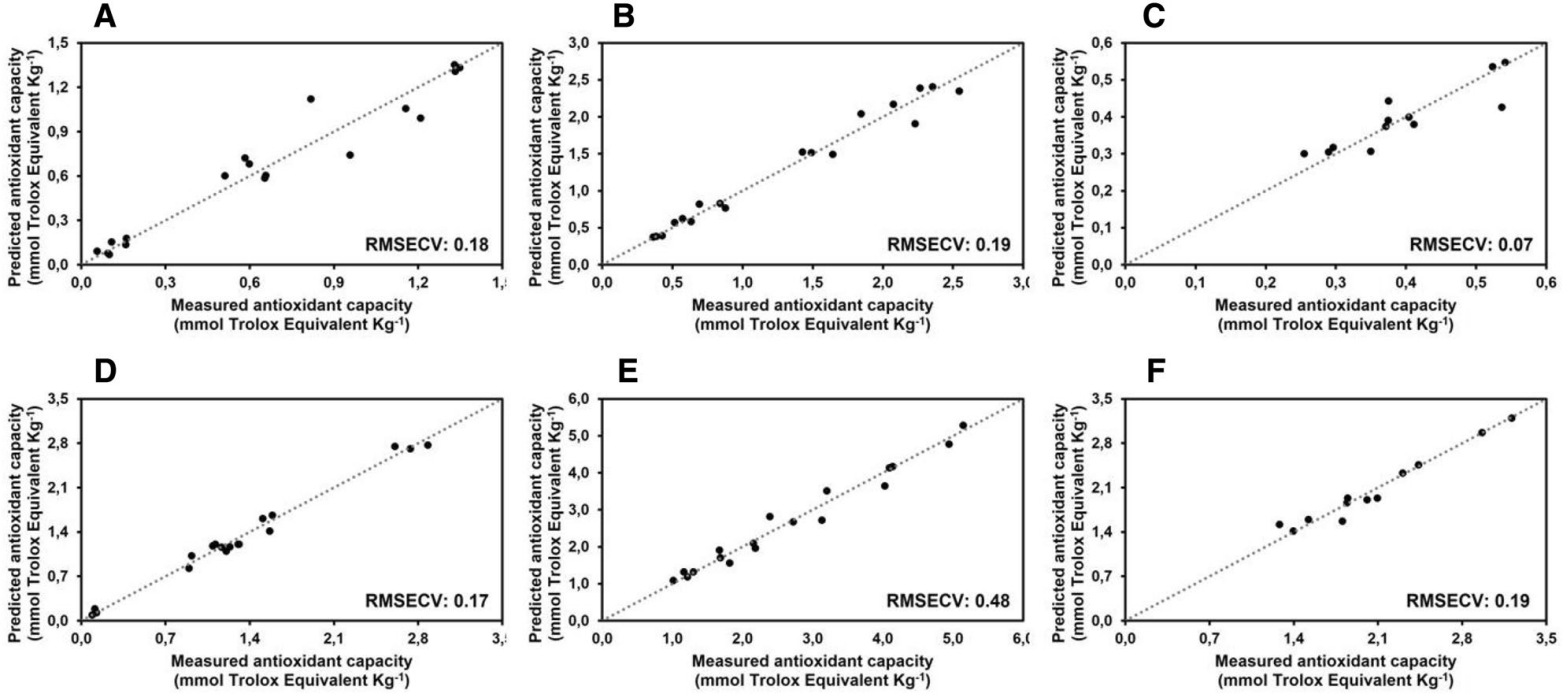
Calculated Partial Last Square model for the prediction of antioxidant capacity on butterhead lettuce (**A–C**) and romaine lettuce (**D–F**). PLS model for the prediction of (**A**) DPPH scavenging capacity of butterhead lettuce leaves; (**B**) FRAP scavenging capacity of butterhead lettuce leaves; (**C**) TEAC scavenging capacity of butterhead lettuce leaves; (**D**) DPPH scavenging capacity of romaine lettuce leaves; (**E**) FRAP scavenging capacity of romaine lettuce leaves; (**F**) TEAC scavenging capacity of romaine lettuce leaves. RMSECV associated with the models are reported.

**Figure 9:**
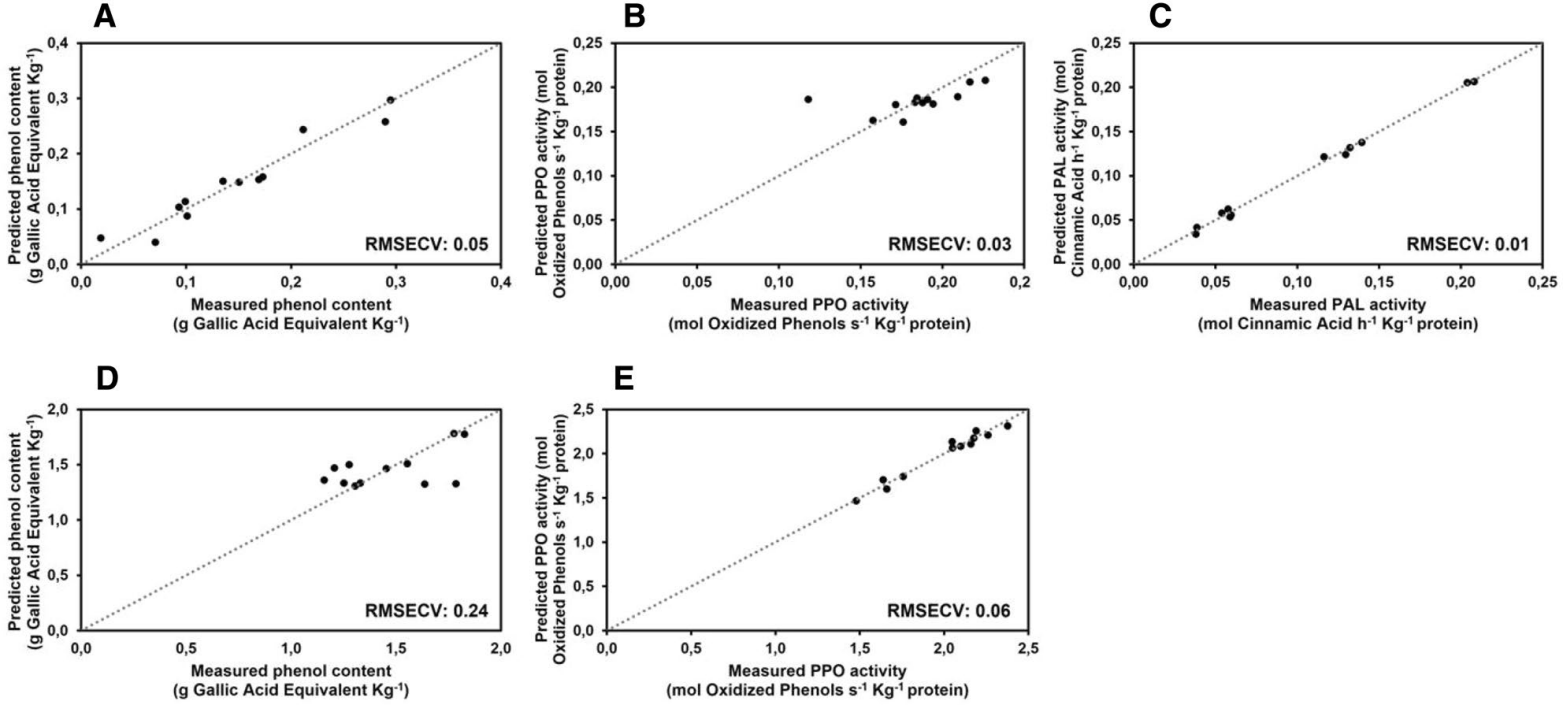
Calculated PLS model for the prediction of phenol metabolism parameters on butterhead lettuce (**A–C**) and romaine lettuce (**D,E**). RMSECV associated with the models are reported.

To verify if metabolic features related to post-harvest disorders can be forecasted by sensor analysis, the measured values of pigment contents, antioxidant capacity, phenol content and PAL and POD activities have been used to calculate Partial Least Square (PLS) regression models. Results are shown in Fig. 7, 8 and 9 where predicted values are plotted versus the expected ones based on sensor analysis. It is evident that the Root Mean Square Error Cross Validation (RMSECV) of pigment contents, obtained using leave-one-out cross-validation technique, were perceptually lower in romaine lettuce (Fig. 7A–C) compared to butterhead lettuce (Fig. 7D–F); the RMSECV for antioxidant capacity parameters are more similar between the two analysed cultivars (Fig. 8), being sometimes slightly higher for butterhead lettuce (Fig. 8B,C) compared to romaine lettuce (Fig. 8E,F); on the other hand, the RMSECV of the detectable parameters related to phenol metabolism were perceptually lower for butterhead lettuce (Fig. 9A,B) compared to romaine lettuce (Fig. 9D,E). These data confirmed that different metabolic features could be related to senescence or post-harvest disorders based on cultivar origin. It also demonstrated that, after an initial validation, the metabolic parameters identified as the most significant markers of senescence and post-harvest disorders can be highly predictable by sensor analysis. This could allow to monitor the quality characteristics of fresh vegetables by a non-destructive approach by processing a huge number of samples.

To the best of our knowledge, this is the first study in which this combined sensor analysis approach was employed and validated for food quality control, relatively to a specific postharvest disorder.

## Conclusion

**Figure 10:**
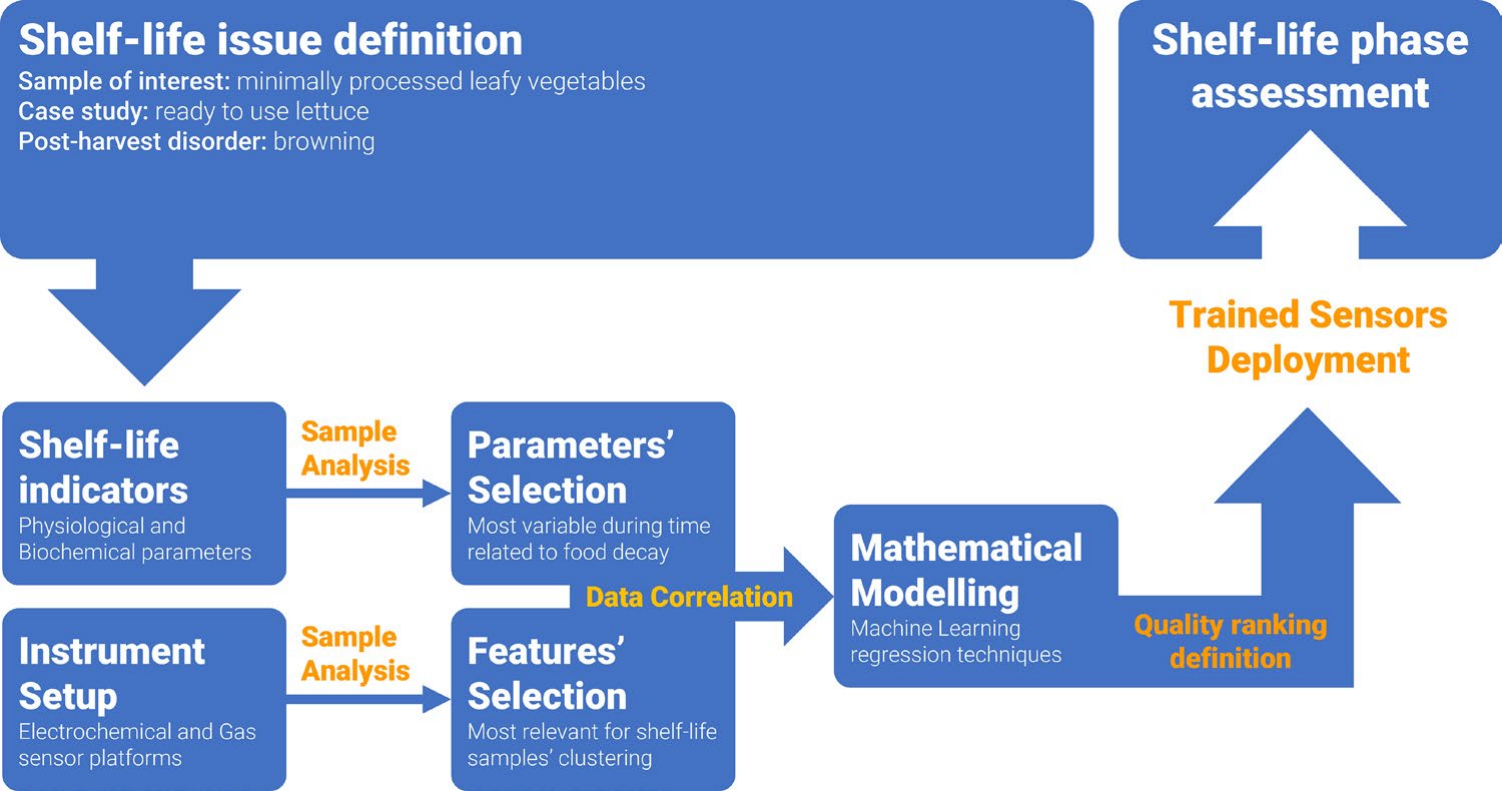
Workflow of the methodological pipeline employed to assess and predict quality attributes of minimally processed plant food over shelf life period.

Here a multiscale approach has been proposed and validated to identify quality features and monitoring overall quality of plant food, possibly minimally processed, over the expected shelf-life period. This approach consisted in the identification of putative (bio)chemical markers, such as antioxidant capacity and phenol metabolism, related to a post-harvest disorder, such as browning, frequently occurring in leafy vegetables under storing conditions. In this context, the ready to use lettuce represented a standardized material to draw the methodological pipeline, which, once validated, can be also used for monitoring nutritional quality of not processed fresh plant food, eventually susceptible to variable post-harvest disorders. The integrated approach revealed that, despite the variable susceptibility of different varieties to post-harvest disorders, the combined analysis of different sensors (gas and electrochemical ones) was able to distinguish samples collected at different days of storage and to predict the (bio)chemical marker trends of the same. Therefore, after a preliminary training, requiring the analysis of various parameters related to the investigated post-harvest disorder and the assessment of the capacity of the sensor platforms to discriminated different shelf-life stages or to describe a quality rank, the analysis can be restrain to the most representative quality marker(s) predicted in not destructive assay by sensor analysis (Fig. 10).

### Supplementary Information

Supplementary Information.

## Data Availability

The data presented in this study are available on request from the corresponding author.

## Abbreviations

MA: Modified atmosphere
TPC: Total phenol content
PPO: Polyphenol oxidase
PAL: Phenylalanine ammonia lyase
PSII: Photosystem II
PhiPSII: Photochemical efficiency of PSII
