# Supplementary material for: Methodological pipeline for monitoring post-harvest quality of leafy vegetables
Source: Sci Rep. 2023 Nov 23;13:20568. doi: 10.1038/s41598-023-47873-4 (PMC10667519; doi:10.1038/s41598-023-47873-4)
Supplement: Supplementary file 1 — Supplementary Information. [file 41598_2023_47873_MOESM1_ESM.doc]

***Supplementary materials***

**METHODOLOGICAL PIPELINE FOR MONITORING POST-HARVEST QUALITY OF LEAFY VEGETABLES**

Tonto T. C.^1^°, Cimini S.^1^°, Grasso S.^2^, Zompanti A.^3^, Santonico M.^2^, De Gara L.^1^, Locato V.^1*^

^1^ Department of Science and Technology for Sustainable Development and One Health, Unit of Food Science and Nutrition, Campus Bio-Medico University of Rome, Via Alvaro del Portillo 21, 00128 Rome, Italy

^2^ Department of Science and Technology for Sustainable Development and One Health, Unit of Electronics for Sensor Systems, Campus Bio-Medico University of Rome, Via Alvaro del Portillo 21, 00128 Rome, Italy

^3^ Department of Engineering, Unit of Electronics for Sensor Systems, Campus Bio-Medico University of Rome, Via Alvaro del Portillo 21, 00128 Rome, Italy

* Corresponding author: email [v.locato@unicampus.it](mailto:v.locato@unicampus.it); Campus Bio-Medico University of Rome, Via Alvaro del Portillo 21, 00128 Rome, Italy; telephone +3906225419468

° These authors equally contributed to this work

**Table S1. Colour changes in butterhead lettuce and romaine lettuce during storage period under air and MA**. L* (from 0 black to 100 white); a* (from -a* green to +a* red); b* (from -b* blue to +b* yellow). Values are means ± SE of at least three biological replicates, each one with three technical replicates. Different letters indicate a statistical difference (p < 0.05) based on one-way ANOVA followed by Tukey test correction. 5* indicates the data referred to the samples collected at day 5 of storage under MA condition.

**
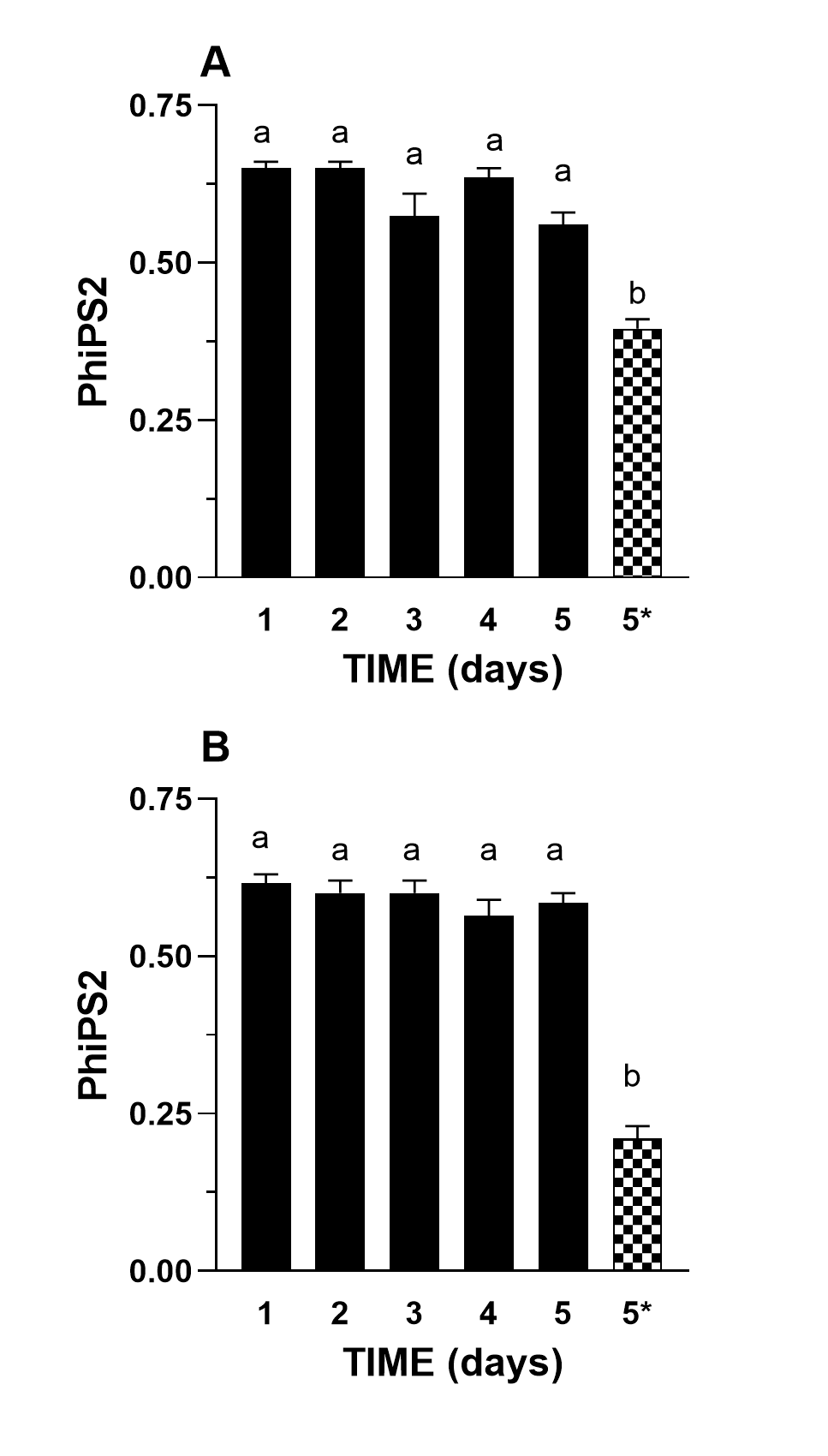
**

**Figure S1 Photosynthetic efficiency of butterhead lettuce (A) and romaine lettuce (B) during storage period under air and MA conditions.** Values are means ± SE of at least three biological replicates, each one with three technical replicates. Different letters indicate a statistical difference (p < 0.05) based on one-way ANOVA followed by Tukey test correction. 5* indicates the data referred to the samples collected at day 5 of storage under MA condition.
